# T-cell number and subtype influence the disease course of primary chronic lymphocytic leukaemia xenografts in alymphoid mice

**DOI:** 10.1242/dmm.021147

**Published:** 2015-11-01

**Authors:** Ceri E. Oldreive, Anna Skowronska, Nicholas J. Davies, Helen Parry, Angelo Agathanggelou, Sergey Krysov, Graham Packham, Zbigniew Rudzki, Laura Cronin, Katerina Vrzalikova, Paul Murray, Elena Odintsova, Guy Pratt, A. Malcolm R. Taylor, Paul Moss, Tatjana Stankovic

**Affiliations:** 1 School of Cancer Sciences, Department of Medical and Dental Sciences, University of Birmingham, Birmingham, B15 2TT, UK; 2 CRUK Centre, Cancer Sciences Unit, University of Southampton, Southampton, SO16 6YD, UK; 3 Department of Pathology, Heart of England Hospital, Birmingham, B9 5SS, UK; 4 School of Biosciences, University of Birmingham, Birmingham, B15 2TT, UK

**Keywords:** CLL, Mouse model, T-cell depletion

## Abstract

Chronic lymphocytic leukaemia (CLL) cells require microenvironmental support for their proliferation. This can be recapitulated in highly immunocompromised hosts in the presence of T cells and other supporting cells. Current primary CLL xenograft models suffer from limited duration of tumour cell engraftment coupled with gradual T-cell outgrowth. Thus, a greater understanding of the interaction between CLL and T cells could improve their utility. In this study, using two distinct mouse xenograft models, we investigated whether xenografts recapitulate CLL biology, including natural environmental interactions with B-cell receptors and T cells, and whether manipulation of autologous T cells can expand the duration of CLL engraftment. We observed that primary CLL xenografts recapitulated both the tumour phenotype and T-cell repertoire observed in patients and that engraftment was significantly shorter for progressive tumours. A reduction in the number of patient T cells that were injected into the mice to 2-5% of the initial number or specific depletion of CD8^+^ cells extended the limited xenograft duration of progressive cases to that characteristic of indolent disease. We conclude that manipulation of T cells can enhance current CLL xenograft models and thus expand their utility for investigation of tumour biology and pre-clinical drug assessment.

## Abstract (precis)

**Summary:** Manipulation of T cells can enhance current CLL xenograft models, expanding their utility for investigation of tumour biology and pre-clinical drug assessment.

## INTRODUCTION

Chronic lymphocytic leukaemia (CLL), a malignancy of mature B cells, is characterised by the dynamic interaction between quiescent cells in the peripheral blood and cells induced to proliferate by microenvironmental stimuli in proliferation centres of lymphoid organs or bone marrow (Damle et al., 2010; Oppezzo and Dighiero, 2013; ten Hacken and Burger, 2014; Zhang and Kipps, 2014). CLL cells resident in proliferation centres are not readily accessible and the complex microenvironmental interactions, including those with antigen-presenting cells and activated T cells (Oppezzo and Dighiero, 2013; ten Hacken and Burger, 2014), are difficult to recapitulate *in vitro*. This seriously hampers the study of CLL biology and limits pre-clinical assessment of novel therapeutic agents. To overcome restrictions of *in vitro* assays, extensive effort has been invested in development of CLL animal models. Currently, there are two principal approaches: transgenic CLL murine models and adoptive transfer of either primary CLL cells or CLL cell lines into immunodeficient mice (Bertilaccio et al., 2013; Bichi et al., 2002; Chen and Chiorazzi, 2014; Kasar et al., 2012; Klein et al., 2010; Santanam et al., 2010).

Transgenic CLL murine models are suitable for assessment of specific genetic events involved in CLL tumourigenesis (Bertilaccio et al., 2011, 2013; Chen et al., 2009a,b; Chen and Chiorazzi, 2014; Hofbauer et al., 2011; Gorgun et al., 2009; Kriss et al., 2012; Reinart et al., 2013; Santanam et al., 2010; Zanesi et al., 2013) but have several limitations. Delayed onset of leukaemia (Bichi et al., 2002; Hofbauer et al., 2011; Klein et al., 2010; Santanam et al., 2010), differing surface expression of human and murine epitopes (Hu et al., 2009; Leskov et al., 2013) and incapability to recapitulate the intratumour CLL clonal diversity that is inextricably linked to both treatment response and tumour progression (Knight et al., 2012; Landau et al., 2013; Schuh et al., 2012) all limit the use of these models for pre-clinical testing of emerging therapies. Consequently, development and optimisation of primary CLL xenografts that could potentially reconstitute these natural elements of human CLL is highly warranted.

Attempts to develop robust primary CLL xenograft models in NOD/SCID mice deficient in T- and B-cell activity often failed as a result of a combination of absence of the correct tumour environment and presence of natural killer immunity in the host (Dürig et al., 2007; Kobayashi et al., 1992; Shimoni et al., 1999). The production of more severely immunocompromised mice [NOD/LtSz-SCID/IL-2γ*^tm1Wjl^*/SzJ (NSG) and Rag2^−/−^γ_c_^−/−^], which further lack natural killer activity, has partially overcome this issue (Bagnara et al., 2011; Bertilaccio et al., 2010; Devereux, 2011; Herman et al., 2013). Recently, the tumour environment has been addressed and two xenograft models generated (Bagnara et al., 2011). The models employed NSG mice in combination with allogeneic supporting cells either to reconstitute the human haematopoietic system with cord blood-derived CD34^+^ stem cells or to supply a component of the microenvironmental stimuli with antigen-presenting CD14^+^ monocytes. Subsequent modifications have been employed in attempts to remove the need for allogeneic cells (Bertilaccio et al., 2010; Herman et al., 2013). These models indicated a role for T cells, particularly CD4^+^, in both the mediation of CLL engraftment (Bagnara et al., 2011; Devereux, 2011; Dürig et al., 2007; Shimoni et al., 1999) and the resultant CLL cell disappearance with concomitant evidence of lethal graft-versus-host disease (GvHD) (Bagnara et al., 2011; Devereux, 2011). However, both of the original NSG models (Bagnara et al., 2011) exhibited limited graft duration, possibly as a result of associated outgrowth of T cells (Bagnara et al., 2011). Thus, in the light of emerging therapies that utilise and modulate T cells and other components of the microenvironment, it is important that the CLL interactions and T-cell repertoire of patients can be faithfully modelled, for sufficient duration, to facilitate optimal pre-clinical *in vivo* assessment.

**TRANSLATIONAL IMPACT**

**Clinical issue**

Chronic lymphocytic leukaemia (CLL) is currently an incurable malignancy of mature B cells, with a heterogenic clinical course and variable response to treatment. It is characterised by the dynamic interaction between quiescent cells in the peripheral blood and cells that are induced to proliferate by microenvironmental stimuli in lymphoid organs or bone marrow. These proliferation sites are difficult to access and the activating stimuli difficult to recapitulate *in vitro*. To address this deficiency, both transgenic and primary CLL animal models have been developed. The former express murine epitopes and cannot recapitulate intratumour clonal diversity; the latter are poorly characterised and of limited duration owing to T-cell outgrowth. These issues seriously hamper the study of CLL biology and limit the preclinical assessment of novel therapeutic agents. A better understanding of the interaction between B-CLL cells, T cells and other microenvironmental factors, and of the impact of this interaction on disease progression, subclonal diversity and treatment response, would direct the identification of novel treatments. Also, patient-relevant *in vivo* models of sufficient duration that are able to recapitulate the subclonal complexity of CLL are an essential component of preclinical drug assessment and can inform tailored treatment regimens.

**Results**

This work provides an in-depth analysis of T cells in primary CLL xenografts and describes a simple adaptation of current models that enables long-term analysis of CLL progression. The authors show, for the first time, that T-cell numbers affect the course of CLL xenografts in alymphoid mice. Specifically, minimisation of T cells, particularly of the CD8^+^ subset, in aggressive samples extended graft duration to that of indolent (non-aggressive) xenografts. The xenograft models retained several biological properties of primary leukaemias, including disease course, T-cell repertoire and microenvironmental interactions (B-cell receptor signalling and T-cell engagement). All these observations were evident in both of the xenograft models assessed, i.e. CLL xenografts generated by injection of either allogeneic umbilical-cord blood-derived cells or allogeneic monocytes.

**Implications and future directions**

This work highlights the importance of T cells in CLL progression. The T-cell minimisation strategy expands the duration of aggressive CLLs, for which there is an urgent need for new treatment regimens. Thus, this study provides a patient-relevant platform to investigate the role of T cells, tumour progression and efficacy of therapeutic agents, including long-term treatment modalities in preclinical settings. In addition, it helps gain insights into various tumour niches, which are difficult to access in patients. Thus, three rapidly developing areas of interest, namely T-cell biology, new treatment regimes and subclonal diversity, will all benefit from this system that recapitulates CLL natural disease and environment.

In this study, we investigated whether cord blood or monocyte-supported CLL xenograft models can recapitulate the CLL biology and T-cell repertoire of patients and whether titration of autologous T cells prior to xenotransplantation can prolong engraftment in a highly immunocompromised host, thereby enhancing the utility of these models. We observed that both models recapitulate the patient CLL and T-cell phenotype and that titration of T cells or the CD8^+^ subset from patients with progressive tumours prior to xenotransplantation can extend the duration of CLL engraftment.

## RESULTS

### CLL xenografts reflect patient-specific CLL features

We employed reported NSG model variants (Bagnara et al., 2011) in the NOD/Shi-SCID/IL-2Rγc^tm1sug^/Jic (NOG) *Mus musculus* Linnaeus 1758 (mouse) strain that were readily available and bred by our establishment to assess whether xenografts can be established for a range of CLL biological subtypes. NOG mice exhibit a similar immunocompromised phenotype to NSG mice and preliminary studies indicated comparable engraftment kinetics in both strains (Bagnara et al., 2011). Mice humanised by allogeneic CD34^+^ umbilical cord cells or supported by allogeneic CD14^+^ monocytes as T-cell allo-stimuli (Bagnara et al., 2011) were injected with 15 CLL peripheral blood mononuclear cells (PBMCs) representing two broad biological CLL types characteristic of indolent and progressive stages of disease (Table S1).

**Fig. 1.**
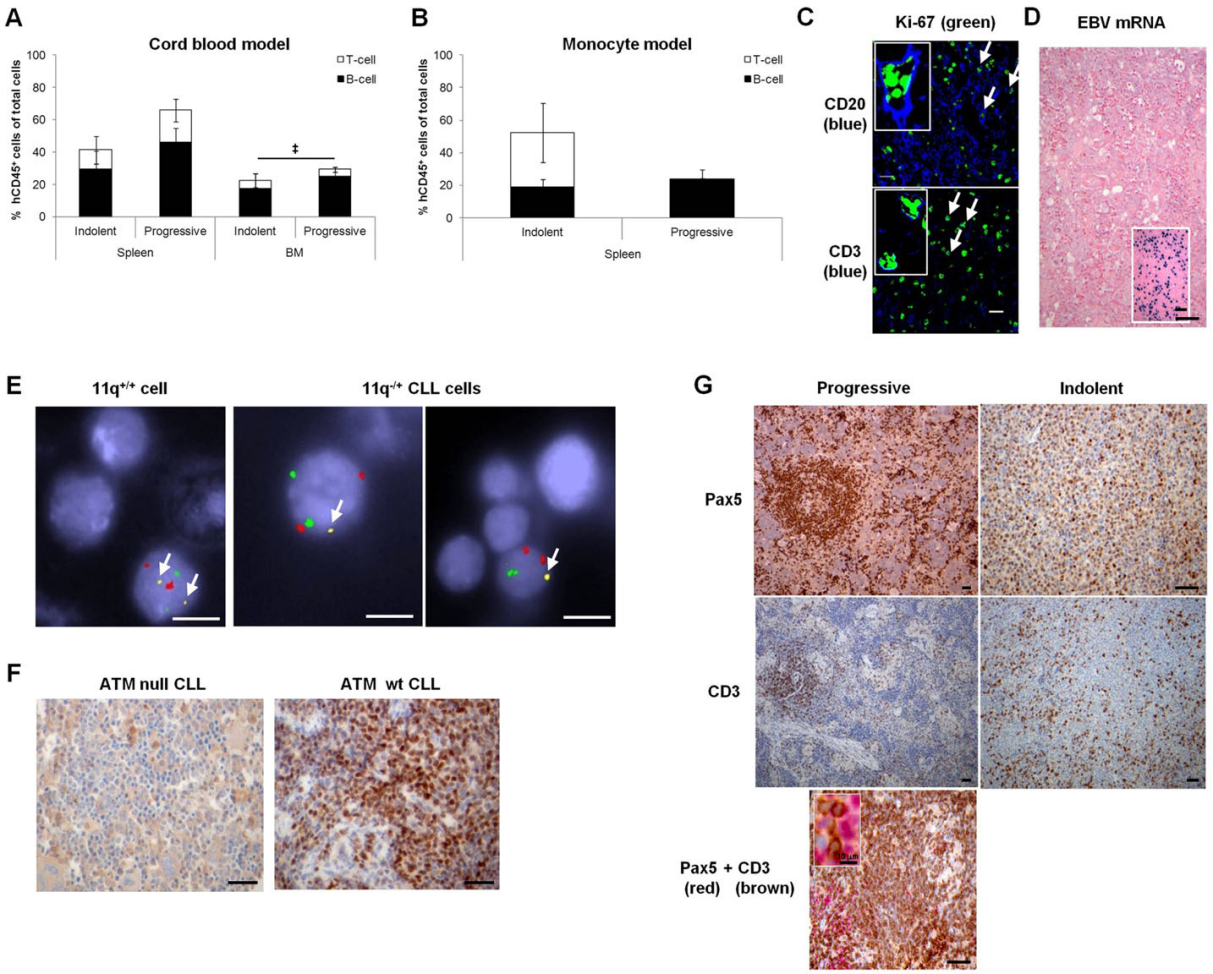
**CLL xenografts of a range of biological subtypes recapitulate patient-specific CLL features.** (A) The proportion of B cells (CD3^−^) or T cells (CD3^+^) that comprise the percentage of engrafted human lymphocytes (hCD45^+^) of total extracted cells at termination, in murine spleen or bone marrow (BM), upon engraftment of indolent (4 CLL, 8 mice, 7.1±0.9×10^7^ PBMC/animal) or progressive (5 CLL, 13 mice, 2.4±0.6×10^7^ PBMC/animal) CLLs in the cord blood model. (B) The proportion of B or T cells that comprise the percentage of engrafted human lymphocytes (hCD45^+^) of total extracted cells upon engraftment of indolent (2 CLL, 3 mice, 6.8±3.2×10^7^ PBMC/animal) or progressive (1 CLL, 4 mice, 0.5±0.0×10^7^ PBMC/animal) CLLs in the monocyte model. The majority of the remainder of the total cell population (hCD45^−^) is of murine origin, possibly with a few human cells expressing undetectable levels of hCD45, including negligible levels of natural killer cells. (C) Representative immunohistological micrographs of CLL engrafted murine spleens depicting expression of Ki67 in proliferating human CD20^+^ B cells and CD3^+^ T cells (examples indicated by white arrows). Images were captured by the Zeiss Zen780 confocal microscope (Cambridge, Cambridgeshire, UK) at 40× magnification prior to article production. (D) Engrafted cells were not derived from normal B cells latently infected with EBV (inset shows positive control). (E) Representative example of 11q^−/+^ cell engraftment identified by FISH. Normal cells contain two copies each of chromosome 10 (red control spots), chromosome 12 (green spots) and 11q (yellow spots, white arrows). The 11q^−/+^ cells in the right hand micrographs only contain a single 11q allele. Cells were visualised at 100× magnification prior to article production. (F,G) Representative immunohistological micrographs of CLL engrafted murine spleens depicting expression of (F) ATM and (G) markers of B-CLL cells (Pax5) and T cells (CD3). (D,F,G) Images were captured by the Leica DMLB microscope with a Leica DFC320 camera (Milton Keynes, Buckinghamshire, UK) at 10× magnification prior to article production. Engraftment levels were compared using one-way analysis of variance (ANOVA) with Tukey post-test (A) or a two-tailed *t*-test (B) and statistical significance denoted by ^‡^*P*≤0.05 (hCD45 engraftment). Scale bars: 20 µm (C), 50 µm (D,F,G), 5 µm (E).

Consistent with NSG mice (Bagnara et al., 2011), support with either CD34^+^ or CD14^+^ cells sustained CLL engraftment, predominantly in murine spleen but also in the bone marrow and blood of all cases (Fig. 1, Fig. S1). In comparison with previous studies (Bagnara et al., 2011; Dürig et al., 2007; Herman et al., 2013), we attained a higher percentage of CLL engraftment. This could relate to differences in experimental conditions, including patient sample engraftment heterogeneity, injection site, single cell isolation method, specificity of CLL markers used for detection, experimental time points and the mouse strain. Nevertheless, the cell numbers retrieved from spleens 12 weeks post-injection (0.75×10^5^ hCD45^+^CD3^−^ versus 0.6×10^5^ hCD45^+^CD19^+^CD5^+^CD23^+^) were comparable (Dürig et al., 2007).

CLL cell proliferation in engrafted spleens was corroborated by Ki-67 positivity and diminishing carboxyflourescein succinimidyl ester (CFSE) intensity (Fig. 1C, Fig. S2). Diminishing CFSE intensity indicated that, at termination, very few non-proliferating CLL cells were present in either the cord blood model or the monocyte model (peripheral blood, 4.1±3.2% versus 0.0±0.0%; spleen, 0.6±0.4% versus 0.3±0.3%; bone marrow, 0.6±0.3% versus 0.0±0.0%; see Fig. S2). Engrafted B cells originated from the malignant rather than the non-malignant compartment, as evidenced by a lack of Epstein–Barr virus (EBV)-encoded RNA (EBER) expression (Fig. 1D) and retention of leukaemic-specific genotypic features (Fig. 1E,F). The architecture observed in patients was reproduced by engrafted CLLs. Progressive CLL cells resembled proliferation centres in lymph nodes of patients and in NSG xenografts (Bagnara et al., 2011), with Pax5^+^ CLL cells surrounded by human CD3^+^ T cells. Indolent CLL B cells were diffusely distributed, consistent with the reduced proliferation levels observed in patients (Fig. 1G) (Giné et al., 2010). Also, in similarity with the scenario in patients (Ghia et al., 2002; Jadidi-Niaragh et al., 2013; Nunes et al., 2012), T cells appear to play a role in CLL progression and, akin to NSG xenografts (Bagnara et al., 2011), the demise of NOG animals was associated with an increasing proportion of T cells (Table S2). In the monocytic model, a minimal proportion of splenic T cells was observed for the progressive CLL, QEo24 (Fig. 1B; Table S2). This could be a feature of this particular CLL, characterised by rapid engraftment, where demise of the animals occurred prior to expansion of splenic T cells but with evidence of T-cell outgrowth in the peripheral blood and bone marrow (Table S2).

**Fig. 2.**
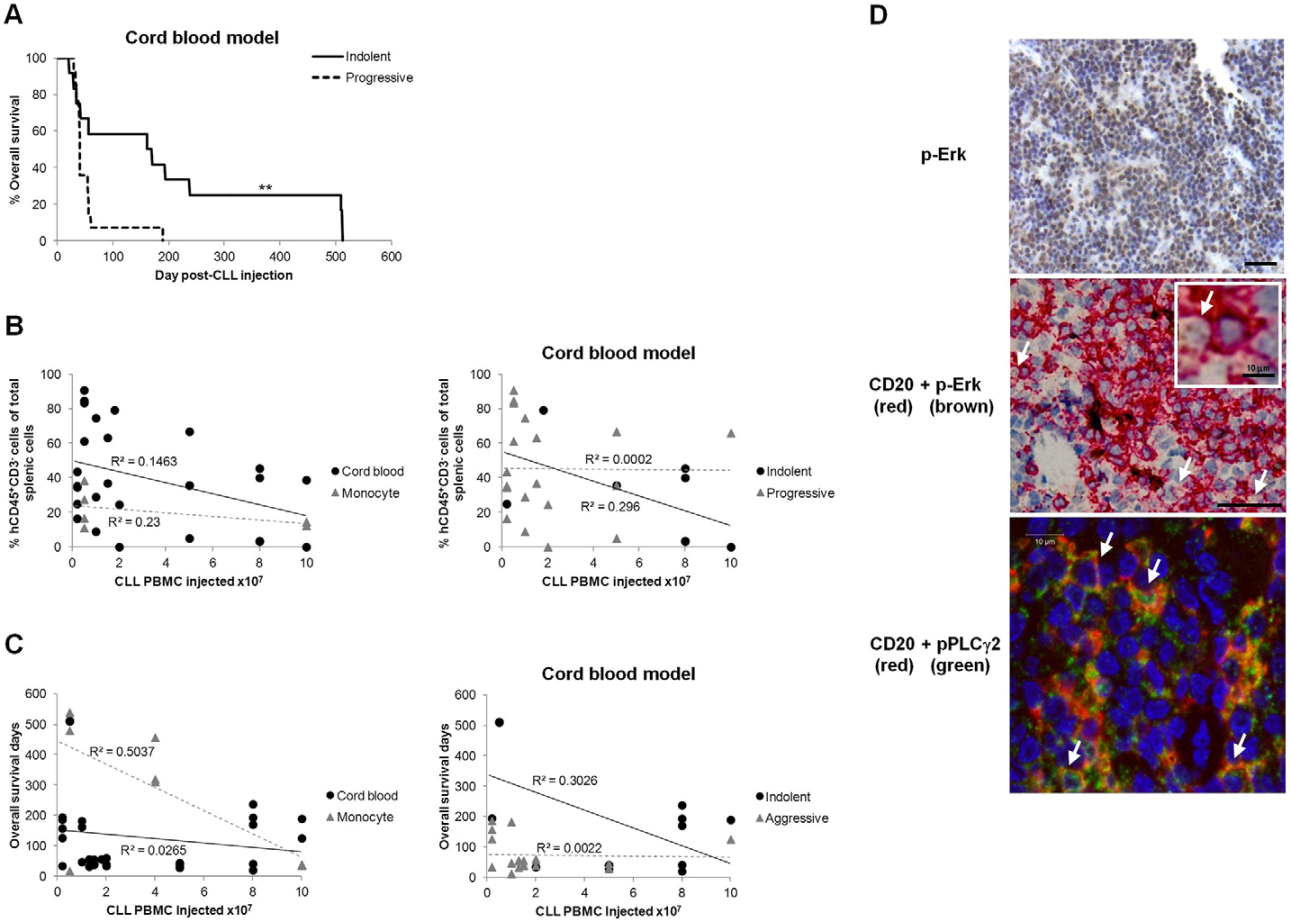
**CLL xenografts recapitulate CLL kinetics and engage the BCR.** (A) Overall survival (OS) of NOG mice after engraftment of indolent (5 CLL, 12 mice, 5.0±1.1×10^7^ PBMC/animal) or progressive (5 CLL, 14 mice, 3.0±0.7×10^7^ PBMC/animal) CLLs in the cord blood model. Correlations between the initial number of injected patient PBMC and (B) the final level of splenic CLL cell (hCD45^+^CD3^−^) engraftment or (C) OS with either CD14^+^ support (monocyte) or prior CD34^+^ cell humanisation (cord blood) for indolent or progressive cases. (D) Representative immunohistological micrographs of CLL engrafted murine spleens depicting expression of p-ERK and pPLCγ2 in CD20^+^ B cells (examples indicated by white arrows) consistent with activation of the BCR signalling pathway. Images of p-ERK were captured by the slide visual Olympus Dotslide microscope (Southend-on-Sea, Essex, UK) at 20× magnification prior to article production. Scale bar: 50 µm. The virtual slide images (VS120) were analysed by OlyVIA (free software, www.olympus.software.informer.com). Images of pPLCγ2 were captured by the Zeiss LSM 510 Meta confocal microscope (Cambridge, Cambridgeshire, UK) at 63× magnification prior to article production. Scale bars: 10 µm. Statistical significance denoted by **P≤0.01.

In contrast to the NSG host (Bagnara et al., 2011), engrafted splenic B cells were present when NOG mice succumbed (Fig. 1). Outgrowing T cells in the cord blood model displayed a variable degree of T-cell chimerism: a mixture of patient and cord blood-derived human T cells (Fig. S3). We observed no evidence of GvHD: engrafted animals did not present with inflammation of the skin or eyes or T-cell infiltration of liver as observed in NSG mice (Fig. S4) (Bagnara et al., 2011). Animal pallor suggested bone marrow failure as a result of replacement of murine haematopoiesis by human leukaemic or T cells as the cause of death. This was evidenced by variable bone marrow replacement with human CD45^+^ cells (10.9-75.4%) in more than 50% of animals (49/80). In the majority of cases (43/49), B cells outnumbered T cells (B cells 8.4-68.5%, mean±s.e.m. 30.9±2.7%; T cells 0.1-22.5%, 4.8±0.8%), but, occasionally (6/49 animals), T cells were the dominant population (T cells 9.2-35.8%, 18.3±4.3%; B cells 1.5-10.2%, 6.2±1.2%). Also, unlike the NSG models (Bagnara et al., 2011), we observed significantly shorter overall survival (OS) of animals engrafted with progressive leukaemias compared with those receiving indolent CLL samples (median survival 41 versus 180.5 days) (Fig. 2A). This was consistent with the higher engraftment levels attained by progressive leukaemias (Fig. 1A,B). Thus, the kinetics of CLL engraftment in NOG mice broadly reflects the disease course in CLL patients.

There was low inter-assay variability in OS and engraftment levels after administration of the same CLL in different experiments (C.E.O., unpublished). Additionally, neither OS nor engraftment levels bore any correlation to the injected number of CLL PBMCs, irrespective of whether xenografts were derived from patients with indolent or progressive disease (Fig. 2B,C). Finally, similarly to CLL cells in lymph nodes (Krysov et al., 2012), phosphorylated ERK1/2 (p-ERK) and phosphorylated-phospholipase Cγ2 (pPLCγ2) were detected in engrafted CLL cells (Fig. 2D). Although not specific for B-cell receptor (BCR) signalling, taken together, detection of these markers was consistent with the notion that the BCR was activated within the engrafted CLL cells (Herman et al., 2014).

### CLL xenografts recapitulate patient T-cell subset proportions

The interaction between T cells and CLL cells is an important aspect of CLL biology (Jin et al., 2010; Kalscheuer et al., 2014; Piper et al., 2011; Riches et al., 2013; Sakuishi et al., 2010; Sumida et al., 2013; Zhou et al., 2011); thus, an understanding of their interdependence in the xenograft model would improve its utility. To address this, we determined the subtypes of engrafted splenic T cells.

**Fig. 3.**
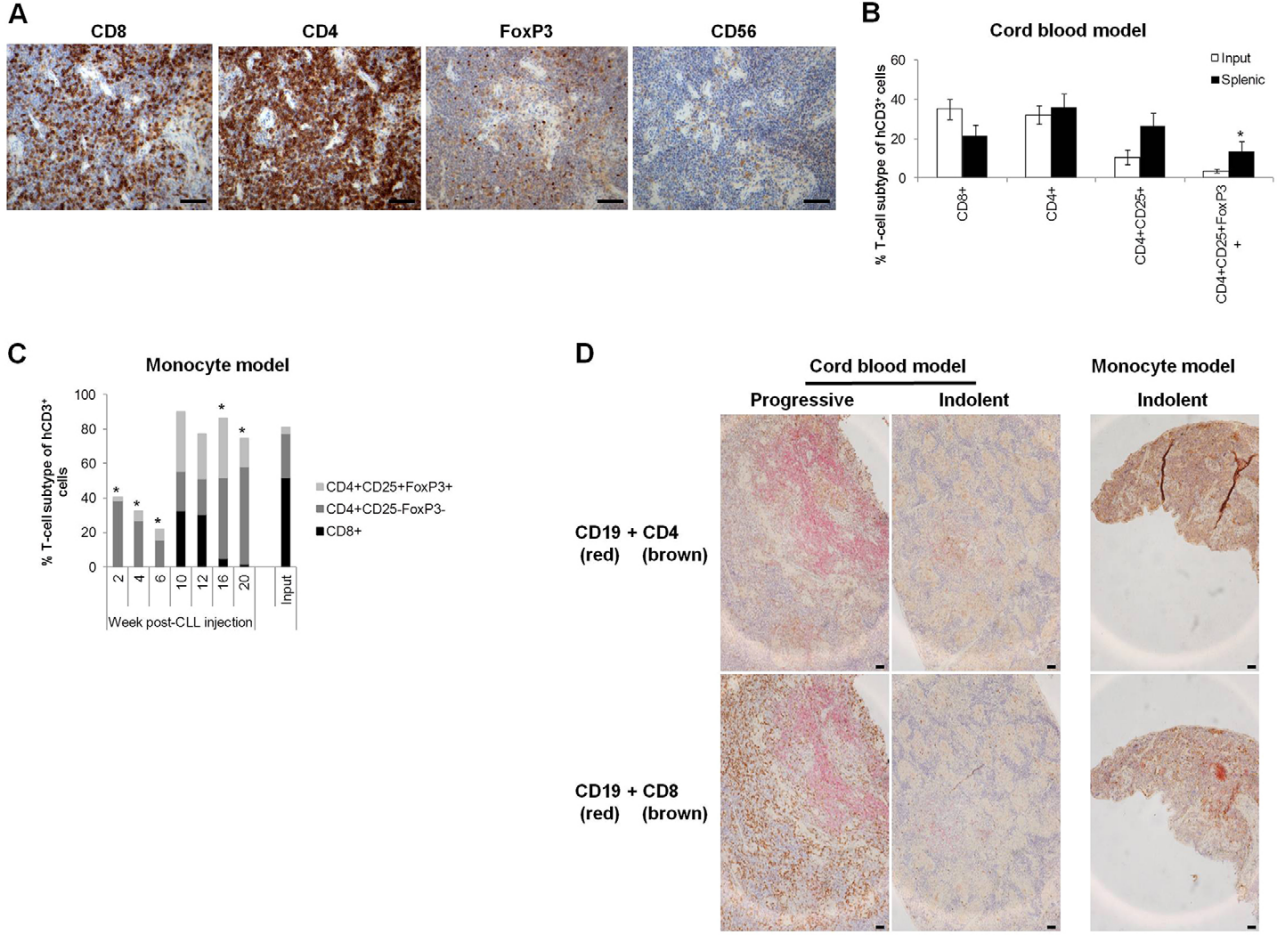
**Engrafted T cells reflect the patient subtype repertoire.** T cells derived from terminally engrafted spleens from the cord blood model recapitulate the T-cell subtype characteristics of CLL cells. (A) Representative immunohistological micrographs depicting engrafted human T-cell subtype marker expression. Images were captured by the Leica DMLB microscope with a Leica DFC320 camera (Milton Keynes, Buckinghamshire, UK) at 10× magnification prior to article production. (B) FACS analysis quantification and comparison of T-cell subsets in xenograft-derived splenic human cells CD8^+^, CD4^+^ and CD4^+^CD25^+^ (9 CLL, 20 mice) and CD4^+^CD25^+^FoxP3^+^ (5 CLL, 5 mice) and in patient samples (input) CD8^+^, CD4^+^ (11 CLL), CD4^+^CD25^+^ (10 CLL) and CD4^+^CD25^+^FoxP3^+^ cells (9 CLL). (C) Engraftment kinetics of the various patient-derived T-cell subsets were followed by analysis of sequentially sacrificed animals. Mice with CD14^+^ monocyte support were injected with PBMCs from one of three CLL patients (*n*=8 mice/CLL). A single animal from each cohort was sacrificed bi-weekly for 20 weeks and splenic cell composition analysed by FACS. Terminal engraftment had not been attained by this time-point. (D) Representative immunohistological micrographs illustrating the differing splenic distribution and contact between engrafted human CD19^+^ CLL cells (red) and either CD4^+^ or CD8^+^ T cells derived from indolent or progressive CLLs. Images were captured by the Nikon Eclipse E400 microscope with a Nikon DS-Fil camera linked to Nikon Digital Sight Capture (Kingston upon Thames, Surrey, UK) at 10× magnification prior to article production. Subtype proportions were compared using a two-tailed *t*-test (B), one-way ANOVA with Dunnett's post-test versus Input or two-way ANOVA with Bonferroni post-test versus Week 2 (C); *P≤0.05 (versus Input). Scale bars: 50 µm.

In the cord blood model, terminally engrafted T cells were predominantly of CD4^+^ subtype, regardless of the biological properties of the CLL and the length of CLL engraftment. The majority of T-cell subsets observed in patients could be detected in CLL xenografts in similar proportions, except for significantly (*P*≤0.05) elevated levels of T-regulatory cells (CD4^+^CD25^+^FoxP3^+^), typically associated with CLL progression (Jadidi-Niaragh et al., 2013) (Fig. 3A,B). Ratios of CD4 to CD8 were within the normal range for CLL (Nunes et al., 2012) and insignificantly altered from that of the patient PBMCs, thus remained characteristic of patient disease status (Fig. S5). The slight discrepancies between patient PBMCs and xenograft spleens were in accordance with observations in the NSG models suggesting that the CD4:CD8 ratio differed between murine peripheral blood and spleen (Bagnara et al., 2011). Thus, irrespective of the T-cell origin (patient-derived, cord blood-derived or a mixture) in the cord blood model (Fig. S3), the patient T-cell subset proportions were retained (Fig. 3B and Fig. S5).

A bi-weekly sequential cull of one animal from both the cord blood (C.E.O., unpublished) and monocyte models revealed that the autologous T-cell repertoire was dynamic and continually changing. The repertoire observed in patients' peripheral blood was gradually acquired in murine spleens within 10-12 weeks, whereas at all other time-points, the proportion of splenic CD8^+^ cells differed significantly (*P*≤0.05) from patient PBMCs. This presented as an initial preponderance of splenic CD4^+^ cells with gradual expansion of T-regulatory cells (CD4^+^CD25^+^FoxP3^+^), followed by expansion prior to a decline in CD8^+^ cells (Fig. 3C). Finally, human CD4^+^ T cells were revealed to be within the splenic proliferation centres surrounded by CD8^+^ T cells for progressive CLL xenografts, whereas a diffuse distribution of both subsets was observed for indolent CLL xenografts (Fig. 3D).

### Engrafted T cells display a dysfunctional phenotype

**Fig. 4.**
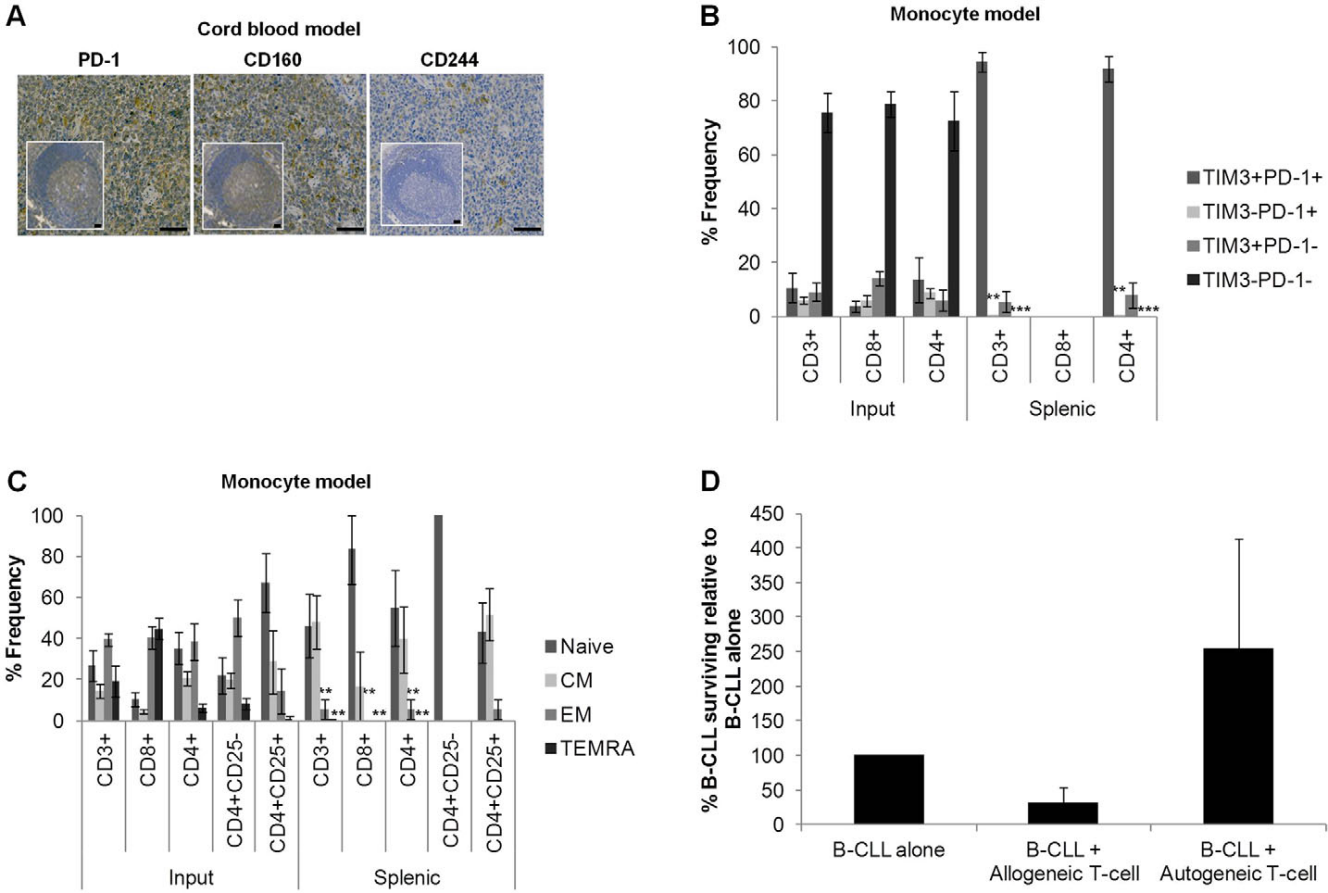
**Engrafted T cells display a dysfunctional phenotype typical of CLL.** (A) Representative immunohistological micrographs depicting expression of the exhausted markers hPD-1, hCD160 and hCD244 in spleens engrafted using the cord blood model in comparison with healthy human tonsils (inset). Images were captured by the slide visual Olympus Dotslide microscope (Southend-on-Sea, Essex, UK) at 20× magnification prior to article production. Scale bars: 50 µm. The virtual slide images (VS120) were analysed by OlyVIA (free software, www.olympus.software.informer.com). (B) FACS analysis demonstrating the T-cell expression of markers of exhaustion (TIM3^+^ PD-1^+^), anergy (TIM3^−^ PD-1^+^), senescence (TIM3^+^ PD-1^−^) and normal function (TIM3^−^ PD-1^−^) (Crespo et al., 2013) in the monocyte model (2 CLL, 5 mice) and patient PBMCs (3 CLL). (C) FACS quantification of naive (CCR7^+^ CD45RA^+^), CM (CCR7^+^ CD45RA^−^), EM (CCR7^−^ CD45RA^−^) and TEMRA (CCR7^−^ CD45RA^+^) human T cells from either injected patient PBMCs (3 CLL) or xenograft-derived splenic cells (3 CLL, 6 mice). (D) Quantification by FACS analysis of CD19^+^ B-CLL cells following co-culture of splenic T cells with B-CLL cells at a ratio of 3:1 reveals a tolerance to the autologous input B cells (*n*=3). Data was normalised to the number of surviving B cells after culture of CD19^+^ B-CLL cells in the absence of extraneous T cells (B-CLL alone). Data were compared using one-way ANOVA with Tukey post-test and statistical significance versus input (B,C) or B-CLL alone (D) denoted by **P≤0.01, ***P≤0.001.

A T-cell exhaustion phenotype previously reported in patients (Riches et al., 2013) was also evident in cord blood-supported xenografts. Of note, the PD-1^+^ expression levels of the input samples were low (13.0-36.3% of CD4^+^; 3.1-14.7% of CD8^+^) but fell within the broad range reported for CLL PBMCs, which spans and encompasses the healthy donor range (Brusa et al., 2013; Gassner et al., 2014; Nunes et al., 2012; Riches et al., 2013; te Raa et al., 2014). However, engrafted splenic cells displayed high expression of the exhaustion markers human (h)PD-1, hCD160 and hCD244 (Fig. 4A). Further assessment utilising the monocyte model, in which all T cells can only be patient-derived, confirmed an exhaustion phenotype (TIM3^+^PD-1^+^) (Crespo et al., 2013; Jin et al., 2010; Sumida et al., 2013; Zhou et al., 2011). However, akin to the CD4:CD8 ratio discrepancies observed in the NSG model (Bagnara et al., 2011), engrafted splenic proportions were skewed towards a significantly (*P*≤0.01) greater proportion of exhausted T cells (TIM3^+^PD-1^+^), with concomitant fewer anergic (TIM3^−^PD-1^+^) and senescent or functional (TIM3^+/−^PD-1^−^) T cells compared with patient PBMCs (Fig. 4B). The observed T-cell phenotype differences between patient PBMCs and engrafted splenic T cells could be a result of their interaction with tumour cells in murine spleens or merely a side effect of xenotransplantation. However, studies using the Eµ-TCL1 transgenic adoptive transfer mouse model have demonstrated that the tumour, rather than transplantation into a secondary recipient, is responsible for the exhausted phenotype of splenic T cells (Gassner et al., 2015; McClanahan et al., 2015).

Also, in contrast with the predominant effector memory (EM) and effector memory RA (TEMRA) phenotypes observed in the peripheral blood of CLL patients (Riches et al., 2013), terminal engrafted splenic T cells bore a predominantly naive or central memory (CM) phenotype with significantly less (*P*≤0.01) EM and TEMRA for all T-cell subsets (Fig. 4C); a repertoire that is indicative of anergy and thus tolerance (Crespo et al., 2013). Thus, in similarity to the scenario in patients, CLL cells appear to influence the T-cell phenotype in CLL xenografts. The phenotypic discrepancies between T cells from patient PBMCs and engrafted spleens could be attributed to the dynamic and varying nature of T-cell interactions in diverse locations and microenvironments (Jin et al., 2010; Sakuishi et al., 2010; Sumida et al., 2013; Zhou et al., 2011).

We speculated that dysfunctional human T cells recovered from the xenografts are unlikely to exhibit anti-CLL activity. Indeed, upon *in vitro* co-incubation of engrafted human splenic T cells with autologous CLL cells, the level of surviving CLL cells increased, albeit not significantly, potentially indicating a protective effect of these T cells. In contrast, the T cells retained limited reactivity towards allogeneic CLL cells (Fig. 4D). This, in combination with absence of GvHD (Fig. S4), is consistent with T-cell anergy (tolerance of self and host with retention of some reactive activity). This concurs with a report indicating tolerance of engrafted human T cells towards murine HLA types and concurrently injected allogeneic human cells as a result of T-cell anergy (Kalscheuer et al., 2014).

Together, these results indicate that engrafted splenic T cells predominantly express exhaustive phenotypic markers. However, features of anergy (proliferation, tolerance of host and autologous cells with retention of limited allogeneic functional activity) and senescence (TIM3^+/−^PD-1^−^) are also evident. Thus, in similarity to cells from CLL patients, engrafted T cells are dysfunctional, composed of co-existing populations in various states and predominantly exhaustive, but also anergic and possibly senescent.

### Controlled depletion of autologous T cells expands the window of CLL engraftment of progressive CLLs for treatment evaluation

It has been reported that complete T-cell depletion prevents engraftment (Bagnara et al., 2011). Thus, we hypothesised that reduction of T cells to a minimal number sufficient to provide growth stimuli for CLL cells could delay the onset of rapid T-cell proliferation and consequently prolong CLL engraftment for treatment evaluation.

**Fig. 5.**
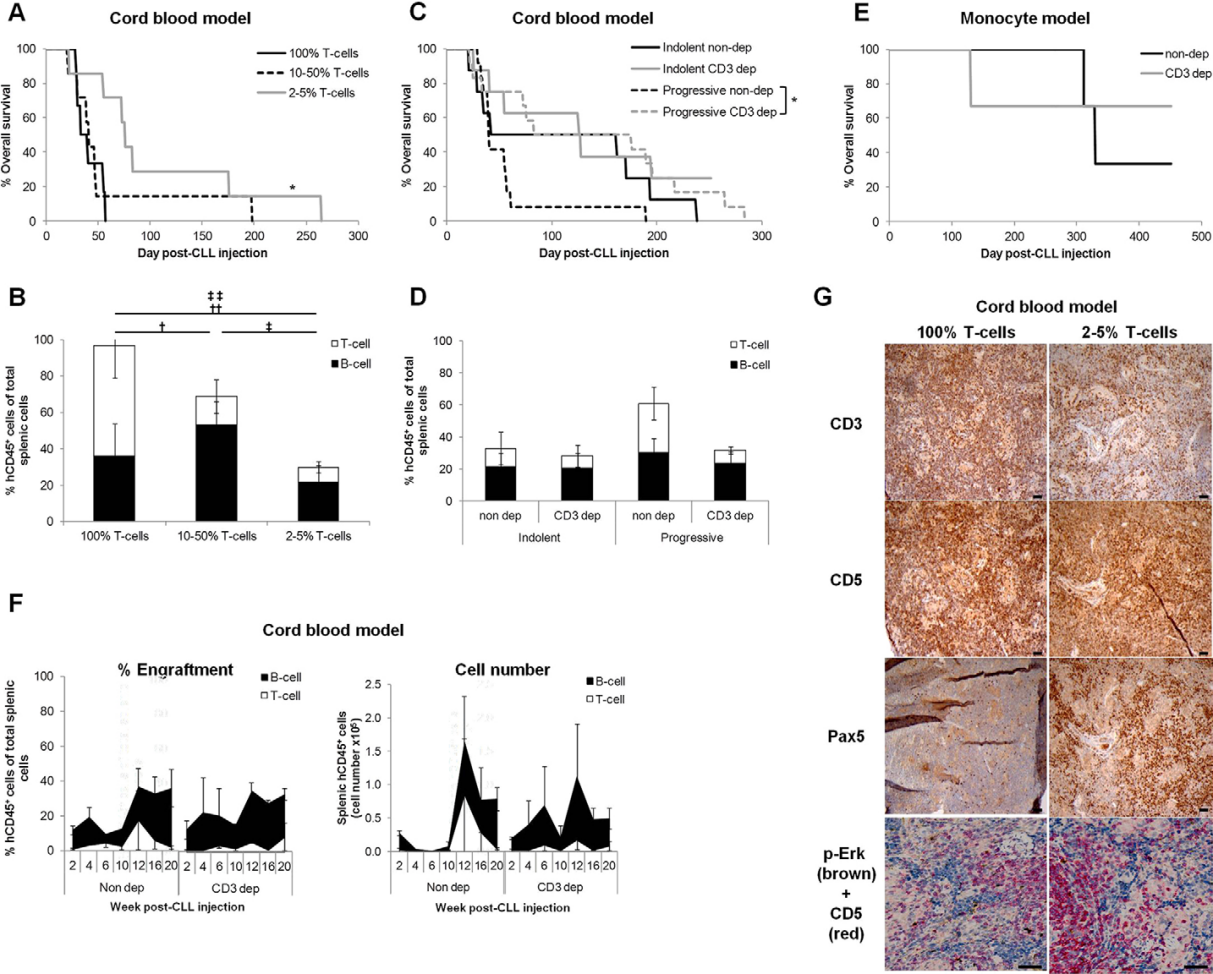
**Controlled depletion of autologous T cells sustains B-cell engraftment and expands the window of CLL engraftment in progressive CLLs.** To determine the effect of T-cell manipulation on CLL engraftment kinetics, mice were injected with CLLs from which the T cells (CD3^+^) had been titred out to various levels. There was no significant variation in the number of injected PBMCs or B cells between any of the groups. (A) OS of animals injected with the same 3 CLL PBMCs containing 100% T cells (18×10^4^ to 230×10^4^ T cells, 6 mice, 3.2±0.8×10^7^ PBMC/animal), 10-50% T cells (4.55×10^4^ to 115×10^4^ T cells, 7 mice, 3.4±0.8×10^7^ PBMC/animal) or 2-5% T cells (0.91×10^4^ to 4.6×10^4^ T cells, 7 mice, 3.4±0.8×10^7^ PBMC/animal) of their initial T cells. (B) The proportion of B cells (CD3^−^) or T cells (CD3^+^), comprising the percentage of engrafted splenic human lymphocytes (hCD45^+^) of total extracted cells, at graft termination of animals administered with CLL PBMCs titred to 100% T cells (2 CLL, 3 mice, 5.0±0.0×10^7^ PBMC/animal), 10-50% T cells (3 CLL, 5 mice, 4.3±0.7×10^7^ PBMC/animal) or 2-5% T cells (3 CLL, 6 mice, 3.8±0.8×10^7^ PBMC/animal) was monitored by FACS in the cord blood model. (C) OS of animals engrafted with five progressive (12 mice, 2.7±0.8×10^7^ PBMC/animal) or four indolent (8 mice, 7.1±0.9×10^7^ PBMC/animal) CLLs in comparison with their T-cell depleted counterparts: CD3 dep progressive (12 mice, 2.5±0.5×10^7^ PBMC/animal, 15±5% T cells remaining) or CD3 dep indolent (8 mice, 6.4±0.8×10^7^ PBMC/animal, 25±10% T cells remaining) CLLs. (D) The proportion of B cells (CD3^−^) and T cells (CD3^+^), comprising the percentage of engrafted splenic human lymphocytes (hCD45^+^) of total extracted cells, at graft termination of mice engrafted with indolent CLLs (4 CLL, 6 mice, 7.8±0.7×10^7^ PBMC/animal), after T-cell depletion (3 CLL, 6 mice, 5.9±1.0×10^7^ PBMC/animal, 30±11% T cells remaining) and progressive CLLs (4 CLL, 8 mice, 3.4±1.1×10^7^ PBMC/animal), depleted of T cells (5 CLL, 11 mice, 2.6±0.6×10^7^ PBMC/animal, 15±5% T cells remaining) in the cord blood model was quantified by FACS analysis. No significant variation was found between any of the groups or between the level of T-cell depletion. (E) The monocyte model was also used to follow the OS of mice engrafted with CLL PBMCs containing 100% (1 CLL, 3 mice, 4.0×10^7^ PBMC/animal) or 2-5% (1 CLL, 3 mice, 4.0×10^7^ PBMC/animal) of patients' T cells. (F) Engraftment kinetics were followed by analysis of sequentially sacrificed animals. Cord blood cell humanised mice were injected with PBMCs from one of two progressive CLL patients with (*n*=8 mice/CLL, 0.2×10^7^ to 2×10^7^ PBMC/animal, 17±12% T cells remaining) and without (*n*=8 mice/CLL, 0.1×10^7^ to 0.3×10^7^ PBMC/animal) prior reduction of patient T cells. A single animal from each cohort was sacrificed bi-weekly for 20 weeks and splenic cell composition analysed by FACS. Terminal engraftment had not been reached by this time-point. (G) Representative immunohistological micrographs of cord blood xenograft spleens with and without prior depletion of T cells, at graft termination: B cells (Pax5^+^, CD5^+^), T cells (CD3^+^, CD5^+^), BCR signalling (dual p-ERK^+^/CD5^+^ stain). Images were captured by the Leica DMLB microscope with a Leica DFC320 camera microscope (Milton Keynes, Buckinghamshire, UK) at 10× magnification prior to article production. Scale bars: 50 µm. Engraftment and input levels were compared using one-way ANOVA with Tukey post-test (B,D), one-way ANOVA with Dunnett's post-test versus Input or two-way ANOVA with Bonferroni post-test versus Week 2 (F). Statistical significance is denoted by *P≤0.05 (A,C), ^†,‡^*P*≤0.05, ^††,‡‡^*P*≤0.01 (B; versus Week 2) († T cell, ‡ hCD45 engraftment).

To test this hypothesis, autologous CD3^+^ T cells were titrated prior to xenotransplantation. Mice injected with 2-5% of patients' initial T-cell population (7-25 T cells per 1×10^4^ B cells) survived significantly (*P*≤0.05) longer than those with either 10-25% or the full complement of T cells (35-667 T cells per 1×10^4^ B cells) (median survival 76, 41 and 37 days; respectively) (Fig. 5A). Prolonged survival of mice injected with T-cell-depleted PBMCs was not a result of differing numbers or variable compositions of injected cells nor lack or loss of CLL engraftment. Input cell number had no impact on OS or engraftment levels (Fig. 2B,C). Also, with the exception of T cells that were manipulated, input compositions did not differ between the cohorts as CLLs were matched between them. The level of B-CLL engraftment was not significantly affected in spleens from terminally unwell animals injected with the same tumours following T-cell depletion (Fig. 5B,D; Table S2). However, titration of T cells to 2-5% significantly reduced the level of T cells and, thus, total lymphocyte (hCD45^+^) engraftment in this cohort (Fig. 5B). The effect of T-cell depletion upon animal survival was most significant (*P*≤0.05) among the short-lived xenografts obtained from progressive CLLs (Fig. 5C,D). Thus, T-cell manipulation extended the disease course of progressive CLL xenografts to that of indolent CLL, indicating an important role of T cells in CLL progression. Confirmation that T-cell manipulation could be used as a universal approach to prolong CLL engraftment, irrespective of the CLL xenograft model, was obtained by application of the same T-cell reduction approach in the monocyte model. Again, a trend towards increased survival upon reduction in T-cell number was observed (Fig. 5E).

Time-courses provided evidence that prolonged OS of engrafted animals following T-cell depletion was not a result of delayed CLL proliferation and that the duration of CLL engraftment was expanded. A bi-weekly sequential cull, over 20 weeks, of paired mice with progressive CLL xenografts, with and without T-cell restriction, revealed no significant difference in splenic CLL engraftment kinetics (Fig. 5F, Fig. S6). However, T-cell restriction delayed the appearance of T cells (Fig. 5F, Fig. S6; Table S2). The fluctuation in T-cell numbers through the time-course highlights the spatio-temporal dynamic nature of their interaction with CLL cells (Fig. 5F). Analogous with the dysfunctional nature of engrafted T cells (Fig. 4), the total number of engrafted B cells populating the spleen did not diminish upon the appearance of T cells (Fig. 5F; Fig. S6, right-hand panels). Also, splenic CLL engraftment was sustained following prior T-cell depletion, as evidenced by fewer T cells present at termination and a concomitant larger B-cell population. CLL cells retained the lymphoma morphology and BCR signalling in T-cell restricted xenografts, indicating that the depletion procedure did not abrogate CLL cell function. The abundance of CLL cells in response to T-cell depletion indicates that the majority of cells engaging the BCR were CLL rather than T cells (Fig. 5G).

We conclude that a reduction in the number of patients' T cells to ≤5% of the initial population prolongs CLL engraftment, particularly for progressive tumours, whilst retaining CLL function and engagement in murine tissues. Our data support a xenotransplantation model where CLL cells only require a restricted number of T cells for their proliferation.

### CD8^+^ subsets influence the kinetics of CLL engraftment

**Fig. 6.**
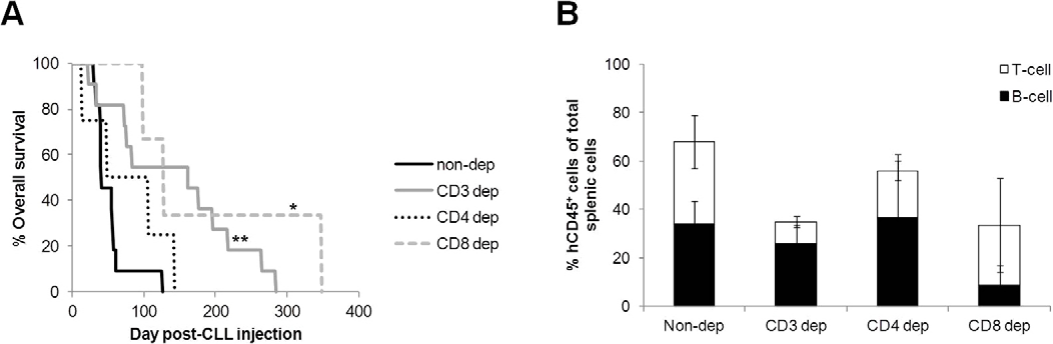
**Specific depletion of the CD8^+^ T-cell subset expands the window of CLL engraftment of progressive CLLs.** (A) OS and (B) the proportion of B cells (CD3^−^) or T cells (CD3^+^) that comprise the percentage of engrafted splenic human lymphocytes (hCD45^+^) of total extracted cells at termination following adoptive transfer of progressive CLLs from which specific T-cell subsets had been depleted in the cord blood model (non-depleted, 4 CLL, 11 mice, 3.0±0.8×10^7^ PBMC/animal; CD3 depleted, 4 CLL, 11 mice, 2.7±0.5×10^7^ PBMC/animal, 12±5% T cells remaining; CD4 depleted, 2 CLL, 4 mice, 6.0±2.3×10^7^ PBMC/animal, 33±10% CD4^+^ cells remaining; CD8 depleted, 2 CLL, 3 mice, 7.3±2.7×10^7^ PBMC/animal, 0±0% CD8^+^ cells remaining). No significant difference was found between the levels of subset depletion. Engraftment and depletion levels were compared using one-way ANOVA with Tukey post-test. Statistical significance is denoted by *P≤0.05, **P≤0.01.

Finally, the interaction between specific T-cell subsets and engrafted CLLs was investigated. The numbers of CD4^+^ and CD8^+^ T cells from PBMCs of patients with progressive disease were reduced prior to xenotransplantation. As observed for CD3^+^ reduction, specific reduction of CD8^+^ cells significantly (*P*≤0.05) prolonged CLL engraftment and OS of engrafted mice (Fig. 6A; Table S2). In contrast, reduction in CD4^+^ cell numbers had no significant impact. Time to onset of T-cell proliferation differed between the various depletion groups and coincided with the demise of the graft, corroborating a link between T-cell outgrowth and overall survival (Table S2). Even depletion of CD3^+^ cells, which incurred a profound extension of OS, only resulted in an insignificant reduction in terminal splenic engraftment levels (Fig. 6B).

## DISCUSSION

The nature of the stimulus that drives T-cell proliferation during CLL xenotransplantation remains unknown. However, we observed significantly faster kinetics of both CLL and T-cell proliferation in progressive CLL xenografts, suggesting that CLL cells from these patients exert a positive influence on T-cell proliferation. Consistent with this notion, our findings indicate that a minimal fraction of patients' T cells (≤5%), as few as 7 T cells per 1×10^4^ B cells, is sufficient to support CLL proliferation. Also, engrafted T cells gradually acquire the patient's T-cell repertoire, both in terms of the subset frequency and dysfunctional phenotype. This phenomenon was irrespective of T-cell chimerism, suggesting that CLL cells have the ability to shape their microenvironment and influence both the phenotype and proliferation of T-cell populations of different origin in diverse microenvironments. Our results suggest that an interaction between CLL cells and the CD8^+^ T-cell subset may be of particular importance as depletion of the CD8^+^ cells prior to xenotransplantation prolongs engraftment of biologically progressive CLLs, extending it to that of indolent CLL xenografts. Further studies are required to determine the temporal dynamics of the engrafted T-cell repertoire in various murine tissue compartments and identify when it most closely resembles that of patients’ lymph nodes.

Our findings are in agreement with previous reports showing that, in patients, CLL cells can modify the T-cell environment (Ghia et al., 2002; Jadidi-Niaragh et al., 2013; Piper et al., 2011; Yang et al., 2009), induce regulatory T-cell proliferation (Gowda et al., 2010) and elevate CD8^+^ T cells during disease progression (Nunes et al., 2012). This supports the notion of a possible feedback loop whereby CLL cells stimulate expansion of regulatory and CD8^+^ T cells, which in turn reciprocate by supporting CLL proliferation. Our findings differ from observations in early CLL models in NOD-SCID mice, which suggested that partial restriction of T cells could enhance CLL engraftment of indolent but not aggressive CLL cases (Shimoni et al., 1999). Also, CLL xenotransplantation of the highly immunocompromised NSG murine host indicated that T cells are indispensable for engraftment of all subtypes of CLL but that depletion of the CD8^+^ subset does not affect engraftment (Bagnara et al., 2011). These incongruences could reflect differences in the animal strain employed and in experimental conditions.

In CLL xenograft models, human T cells serve two functions: to enable CLL engraftment and to support CLL proliferation. To distinguish between these two roles it would be of interest to manipulate specific T-cell subtypes, post-engraftment, by administration of appropriate antibodies. In addition, combination with a more in-depth characterisation of the T-cell repertoire would greatly enhance our knowledge of their role in CLL biology and therapeutic response. It would be of particular interest to investigate the role of PD-1-expressing T cells in CLL, given the potential for treating multiple myeloma by targeting PD-1 (Atanackovic et al., 2014) and the promising clinical response of solid tumours to anti-PD-1 treatment (Topalian et al., 2012). Nevertheless, the conclusion from this study suggests that CLL xenografts recapitulate the T-cell repertoire observed in patients and therefore represent a useful tool for studying the effects of a wide range of compounds, including the most recently developed immunotherapies.

The duration of a CLL xenograft is a crucial component of *in vivo* assessment of various targeted treatments. The cytotoxic effect of compounds such as DNA damaging agents or antibodies directed against CLL cells can be demonstrated in a relatively short period of time. However, the impact of agents that rely on gradual accumulation of DNA damage (DNA repair inhibitors) or those that require prolonged inhibition of pro-survival signalling (BCR inhibitors) demand extended duration of the CLL graft. Additionally, xenograft duration could be crucial during assessment of drug impact at the subclonal level, particularly to measure eradication of a specific, refractory CLL subpopulation.

In this study, we show that xenotransplantation of primary CLL cells in a highly immunodeficient host can be improved by minimising the number of autologous T cells. Profound depletion of autologous T cells expanded the window of engraftment for progressive CLL tumours without affecting the CLL phenotype. Markers consistent with BCR signalling were retained in xenografts, irrespective of T-cell depletion, and proliferation of CLL subclones with specific cytogenetic abnormalities such as 11q deletion was unaffected by limiting T cells (C.E.O., unpublished). Thus, xenotransplantation with restricted autologous T-cell numbers has the potential to enhance the application of CLL xenograft models. It is tempting to speculate that such enhanced kinetics of xenotransplantation could provide an attractive model for studying the biology of primary CLL progression, an advantage that is currently provided only by transgenic murine CLL models. The issue remains that these models, particularly the cord blood xenografts, are imperfect artificial CLL systems incorporating allogeneic cells. Nevertheless, they still provide a more natural environment than can be modelled either *in vitro* or in transgenic murine models.

Although the retention of the 11q and ATM genotypes and lack of latently EBV-infected healthy B cells indicate that the majority of B cells in these xenografts were tumour-derived, it is plausible that in similarity to T cells, B-cell chimerism could occur in the cord blood model (Bagnara et al., 2011). Further studies are necessary to address this possibility. To some extent, the confounding effect of two hematopoietic systems can be surmounted by utilisation of the monocyte model or CFSE labelling of CLL PBMCs. However, as CFSE labelling declines with each cell cycle this approach is only applicable in short-term studies. Alternatively, distinguishing between the two human haematopoietic systems can be achieved by use of a CLL-specific marker, such as the application of mismatching HLA types of CLL patients and cord blood cells. We believe further optimisation of the variants of CLL xenotransplantation is highly warranted because, despite their weaknesses, each model has the potential for a very specific application. The allogeneic cord blood humanisation model possesses an advantage in addressing the impact of new compounds in the context of the normal human hematopoietic system as a result of its ability to simultaneously monitor their effect on both human non-tumour and leukaemic cells. The allogeneic monocyte support model provides an opportunity to address immunological aspects of CLL biology.

In summary, we show that duration of CLL xenografts can be tailored by manipulation of autologous T cells. This adaptation broadens their utility, particularly for progressive CLL cases, enabling research into the T-cell biology of CLL and assessment of the efficacy of novel treatments, including immunotherapies and those that target the interaction of CLL with the microenvironment. One can envisage a scenario where, by using such a personalised CLL xenograft model, together with new methodologies such as next generation sequencing and single cell analysis, the best combination of compounds targeting different CLL subclones can be easily designed.

## MATERIALS AND METHODS

### Peripheral blood and cord blood samples

CLL and fresh umbilical cord blood samples were collected from local hospitals. Analysis of *IGHV*, *ATM* and *TP53* status was performed as described previously (Skowronska et al., 2012; Stankovic et al., 2002). Blood samples for isolation of CD14^+^ monocytes were donated by healthy volunteers. Studies were approved by the UK National Research Ethics Service Committee West Midlands – Solihull and performed in accordance with local ethical guidelines. Written informed consent was obtained from all patients and healthy donors.

### Cell separation and CFSE staining

PBMCs were enriched for CD14^+^ cells using RosetteSep™ Human Monocyte Enrichment Cocktail (STEMCELL, Manchester, Greater Manchester, UK). PBMC or umbilical cord blood cells were enriched for CD34^+^ or CD3^+^ cells or depleted of CD3^+^, CD4^+^, CD8^+^ or CD25^+^ cells by the magnetic cell sorting system (MACS) (Miltenyi Biotec, Bisley, Surrey, UK). PBMC or T cells were labelled with CFSE as required (Sigma-Aldrich Ltd, Gillingham, Dorset, UK). All procedures were performed according to the manufacturer's instructions.

### Xenotransplantation and treatment

Animal studies were approved by the institutional ethics committee and animals were treated in accordance with UK Home Office guidelines.

Primary CLL xenografts were generated following intravenous (i.v.) injection of 0.1×10^7^ to 10×10^7^ CFSE-labelled (10 µM) patient PBMC cells into γ-irradiated (1.25-2.5 Gy) NOG mice (5-9 weeks old) (in-house) either upon evidence of engraftment (1% hCD45^+^ cells) of pre-injected 1×10^5^ umbilical cord CD34^+^ cells in murine peripheral blood (cord blood model) or concurrently with 1×10^5^ allogeneic CD14^+^ cells (monocyte model) (Bagnara et al., 2011).

### Fluorescence-activated cell sorting (FACS) analysis

Single cell suspensions were labelled with various combinations of the following fluorescently conjugated antibodies: murine (m)CD45, hCD45, hCD19, hCD3, hCD8, hCCR7, hCD45-RA, hTIM3, hCD38, hPD-1 (eBioscience, Hatfield, Hertfordshire, UK), hCD4, hCD25 and hFoxP3 (BD Biosciences, Oxford, Oxfordshire, UK). Analysis was carried out using an LSRII with FACS Diva software (BD, Oxford, Oxfordshire, UK). In some cases, CountBright beads (Invitrogen, Paisley, Renfrewshire, UK) were used to obtain absolute cell counts.

### Fluorescence *in situ* hybridisation (FISH)

Several splenic regions were touched onto slides coated with poly-L-lysine, fixed and air-dried. The following FISH probes were applied: CEP10, CEP12 (Cytocell, Cambridge, Cambridgeshire, UK) and 11q (Abbott Molecular, Maidenhead, UK). Hybridisations were performed with 2 min denaturation at 73°C and 16 h incubation at 37°C on a HyBrite (Abbott Molecular) and subsequent application of 4′6-diamidino-2-phenylindole (DAPI) (Cytocell, Cambridge, Cambridgeshire, UK). Cells (>200/sample) were visualised on an Olympus BX50 microscope (Southend-on-Sea, Essex, UK).

### Immunohistochemistry

Paraffin-embedded sections (5 µm) were stained with haematoxylin and eosin (Sigma-Aldrich) and immunophenotyped using anti–human antibodies against CD5, CD3, CD19, CD56 (Leica, Milton Keynes, Buckinghamshire, UK), Pax5 (Thermo-Scientific, Loughborough, Leicestershire, UK), Ki67, CD3, CD8 (Dako, Ely, Cambridgeshire, UK), ATM (in-house), CD4, FoxP3, pPLCγ2 (Abcam, Cambridge, Cambridgeshire, UK), CD20 (Spring Bioscience, Pleasanton, CA, USA), p-ERK (Cell Signaling, Noston, MA, USA), PD-1, CD160 (Biorbyt, Cambridge, Cambridgeshire, UK), CD244 (Novus Biologicals, Cambridge, Cambridgeshire, UK) and an antibody against EBV transcript (Roche, Burgess Hill, West Sussex, UK), following antigen retrieval with 10 mM citrate buffer (Sigma-Aldrich). Where necessary, a mouse on mouse basic kit (Vector Laboratories, Peterborough, Cambridgeshire, UK) was used according to the manufacturer's instructions. For dual-labelling of CD20 with pPLCγ2, after antigen retrieval the following reagents were sequentially applied, being washed with 0.1% PBS-Tween-20 (Sigma-Aldrich) after each application: blocking agent for 10 min (Vector Laboratories), 1:500 CD20 antibody for 1 h and goat anti-rabbit HRP-labelled IgG for 10 min (PerkinElmer, Waltham, MA, USA). Final visualisation was with Opal™ cyanine 3 fluorophore (PerkinElmer). A second round of antigen retrieval and staining was performed with pPLCg2 antibody (1:500) and Opal™ fluorescein visualisation, followed by counterstaining of slides with DAPI (Life Technologies, Paisley, Renfrewshire, UK).

### Microsatellite analysis

DNA was extracted using a Flexigene kit (Qiagen, Manchester, Greater Manchester, UK) and tested by multiplex PCR using 16 fluorescently labelled primer sets (Nikolousis et al., 2013). Fluorescently labelled products along with size standards were run on an ABI 3130 (PE Applied Biosystems, Warrington, Cheshire, UK).

### Cytotoxicity assay

Samples were enriched for B-CLL cells or xenograft-derived splenic T cells via negative or positive selection with CD3^+^ MACS beads (Miltenyi Biotec). CFSE-labelled (0.5 µM) splenic T cells were co-cultured with B-CLL cells (1×10^5^/well) at a ratio of 3:1 in RPMI media (Sigma-Aldrich) containing 10% fetal calf serum (Sigma-Aldrich) and 10 U/ml recombinant human IL-2 (PeproTech EC Ltd, London, Greater London, UK). After 24 h, B-CLL number was quantified by FACS analysis (anti-hCD19, hCD3 and hCD5; eBioscience, Hatfield, Hertfordshire, UK) with CountBright beads (Invitrogen) (Fox et al., 2010; Hudecek et al., 2010).

### Statistical analysis

Overall survival was compared by Kaplan–Meier curves and Mantel–Cox log rank comparison with Gehan–Breslow–Wilcoxon correction test. Engraftment levels were compared using an appropriate test for the dataset (see figure legends). Statistical analysis was performed using GraphPad Prism v6 (GraphPad Software, San Diego, CA, USA). Data are presented as mean±s.e.m. (standard error of the mean).
